# Developing efficient heavy-atom-free photosensitizers applicable to TTA upconversion in polymer films†
†Electronic supplementary information (ESI) available: Synthetic procedures and characterization data. CCDC 1400097. For ESI and crystallographic data in CIF or other electronic format see DOI: 10.1039/c5sc03245h


**DOI:** 10.1039/c5sc03245h

**Published:** 2015-11-09

**Authors:** Jiang Peng, Xinyan Guo, Xinpeng Jiang, Dahui Zhao, Yuguo Ma

**Affiliations:** a Beijing National Laboratory for Molecular Sciences , Centre for Soft Matter Science and Engineering , Key Lab of Polymer Chemistry & Physics of the Ministry of Education , College of Chemistry , Peking University , Beijing 100871 , China . Email: ygma@pku.edu.cn ; Email: dhzhao@pku.edu.cn ; Fax: +86-10-62751708

## Abstract

Heavy-atom-free triplet photosensitizers are developed by harnessing the thermally active triplet state of carbazolyl dicyanobenzene (CDCB) derivatives and applied to realize visible-to-ultraviolet photon upconversion (UC) *via* triplet–triplet annihilation (TTA). Demonstrating an annihilator-appending strategy, the designed sensitizers effectively realize TTA UC in polyurethane films with 2,7-di-*tert*-butylpyrene (DBP) as the annihilator/emitter. The covalently tethered DBP to CDCB is proven critical for achieving the superior sensitizing and UC performance in the solid matrix, essentially by suppressing the reverse ISC and more effectively transferring triplet excitons to free emitters.

## Abstract 2

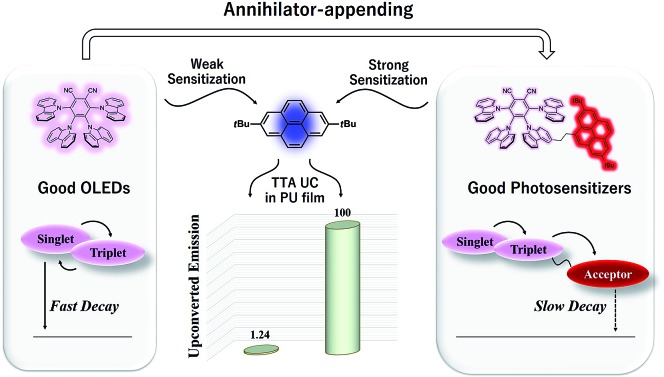
Heavy-atom-free triplet photosensitizers are developed and visible-to-ultraviolet photon upconversion is realized *via* triplet–triplet annihilation.

## Introduction

Triplet photosensitizers that transfer triplet energy to other systems with intrinsically low triplet-state quantum yields^1^ are important to many applications, such as photochemical reactions,^2^ photodynamic therapy,^3^ and photon upconversion (UC) by triplet–triplet annihilation (TTA).^4^ To attain spin–orbital coupling enhanced intersystem crossing (ISC), most of the triplet photosensitizers reported so far contain heavy atoms such as Ir, Pt, Pd, Re, Os, Ru, I, and Br. Organic triplet photosensitizers without heavy atoms are less well known,^5^ mainly due to the typically low ISC efficiency of common organic compounds. However, recent studies have demonstrated that organic molecules with a small singlet–triplet energy gap (ΔE_ST_) may possess increased first-order mixing of the singlet and triplet states and thus produce highly efficient spin conversion in the absence of heavy atoms.^6^ Notably, a small ΔE_ST_ also enables rapid reverse ISC and thermally activated delayed fluorescence (TADF) is thus produced. Such TADF molecules thus have great value in OLEDs as they are capable of exploiting triplet excitons. We, however, speculate that such small ΔE_ST_ induced triplet states may as well be useful in developing triplet photosensitizers. Here we report a series of heavy-atom-free organic photosensitizers designed using such tactics so as to demonstrate their first application in realizing TTA UC in a polymer matrix. Polyurethane is selected as a hosting matrix to conduct TTA UC as it possesses good transparency, high flexibility (low *T*_g_) and suitable mechanical properties (see ESI†).^7^

By virtue of the prominent advantage of utilizing a non-coherent, low-power (mW cm^–2^) light source,^4^ TTA UC is envisioned to have a plethora of potential applications, such as solar energy conversion,^8^ white light emission,^9^ photocatalysis,^10^ and bioimaging.^11^ Ever since Parker and Hatchard first discovered the phenomenon of TTA UC in the 1960s,^12^ various sensitizer and emitter pairs have been shown to be capable of establishing TTA UC in solution.^13^ Yet, achieving this as an efficient process in the solid state still remains a challenging goal, especially to realize practical applications of this technique, considering the ease of device fabrication and processing with solid materials.^14^–^16^ The main obstacle lies in the fact that in solid hosts the molecular mobility is severely restricted and molecular collisions are greatly suppressed, but the triplet sensitization step in TTA UC sensitively relies on such collisional interactions. Consequently, most previously studied solid-state TTA UC systems employed relatively high doping concentrations of emitters and fairly expensive metal-containing sensitizers such as octaethyl porphyrins and ruthenium(ii) complexes with long triplet lifetimes.^17^ Most recently, Baldo *et al.* reported a solid-state upconversion system sensitized by a TADF dye.^18^ Here, to demonstrate the high competency of our metal-free sensitizing system, we show that efficient TTA UC can be achieved with these newly designed sensitizers even in a diffusion-limited polymer matrix.

**Chart 1:**
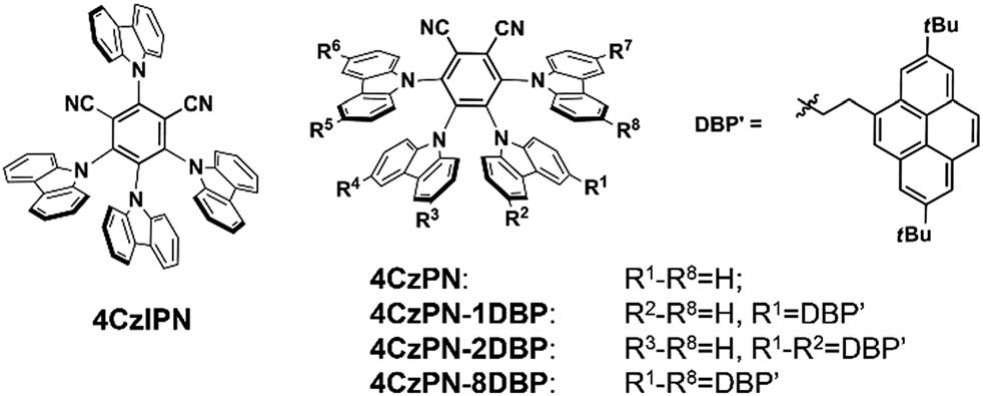
Chemical structures of CDCB-derived heavy-atom-free sensitizers for TTA UC.

We first selected a pair of TADF molecules (4CzPN and 4CzIPN, Chart 1) derived from carbazolyl dicyanobenzene (CDCB) developed by Adachi *et al.*6*a* to examine their triplet sensitizing ability. Our TTA UC experiments show that both 4CzPN and 4CzIPN are reasonably good photosensitizers in solution, as evidenced by the clearly detectable upconverted emission from DBP (2,7-di-*tert*-butylpyrene) upon mixing with 4CzPN or 4CzIPN in toluene (Fig. S6†). An UC quantum yield of 4.4% is reached with 4CzPN.

Nonetheless, the rapid reverse ISC (T_1_ to S_1_) and resultant TADF inevitably presents significant competition to the intermolecular triplet–triplet energy transfer (TTET) between the sensitizer (4CzPN/4CzIPN) and emitter (DBP), particularly under the diffusion limited conditions. Consequently, in the PU matrix, a much less desirable sensitizing performance is observed with 4CzPN and 4CzIPN, rendering hardly detectable UC emission. To improve the intermolecular TTET efficiency in PU films, we then designed three new molecules, which incorporated various numbers of DBP units covalently tethered to 4CzPN *via* ethylene linkers (Chart 1).^19^ These covalently linked DBP are expected to serve as triplet energy acceptors and enable intramolecular TTET, which can effectively compete with the reverse ISC and suppress TADF, thereby favoring the sensitizing process and TTA UC.

## Results and discussion

**Fig. 1:**
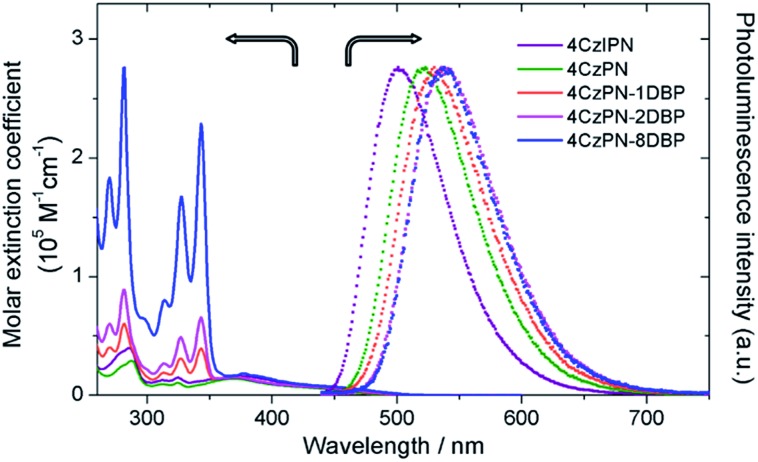
UV-vis absorption spectra (solid) in CH_2_Cl_2_ and normalized photoluminescence spectra (dotted) in toluene (5 μM, excited at 380 nm).

The UV-vis absorption and photoluminescence spectra of all the studied metal-free sensitizers are shown in Fig. 1. The broad peaks around 380 nm are characteristic of charge-transfer (CT) absorptions from CDCB moieties, which tail to about 500 nm, allowing for visible-light excitation used in TTA UC. All three [DBP]-functionalized compounds show additional absorptions from the pyrene units in the UV region with clear vibronic features, in addition to slightly red-shifted CT bands. The photoluminescence spectra of 4CzPN–*n*DBP also show evident red shifting upon [DBP] attachment. Relevant photophysical data are summarized in Table 1. In deaerated toluene, 4CzIPN and 4CzPN show strong fluorescence emissions with two largely differing lifetimes. The longer lifetime components are very sensitive to dissolved oxygen, confirming their TADF identity. The emission quantum yields of the three DBP-tethered analogues are substantially lowered compared to that of 4CzPN, even under N_2_-saturated conditions. The longer lifetime components are not detected, and the emission intensity is relatively less sensitive to oxygen (Table 1). These results are all consistent with the proposition that the triplet exciton generated by the [4CzPN] moiety is intramolecularly transferrable to the attached [DBP], and thus the reverse ISC and TADF are considerably reduced.

**Table 1:** Photophysical data of metal-free sensitizers^*a*^

	*λ*_em_ ^*b*^ [nm]	*τ*_em_ ^*c*^	*τ*_T_ ^*d*^ [μs]	*Φ*_em_ ^*e*^ [N_2_/air]
4CzIPN	501	14 ns (22%)/4.5 μs (78%)	—	0.87/0.26
4CzPN	523	11 ns (17%)/11.6 μs (83%)	8.7	0.74/0.13
4CzPN–1DBP	530	17 ns	263	0.22/0.17
4CzPN–2DBP	536	23 ns	281	0.31/0.24
4CzPN–8DBP	538	9 ns^*f*^	365^*f*^	0.10/0.09

^*a*^Data are measured in deaerated toluene at room temperature.

^*b*^Emission maximum wavelength.

^*c*^Photoluminescence lifetimes.

^*d*^Excited state lifetimes measured using nanosecond transient absorption.

^*e*^Photoluminescence quantum yields measured in N_2_- or air-saturated toluene at room temperature using [Ru(bpy)_3_]Cl_2_ as the standard.^20^

^*f*^Averaged lifetimes from fitting bi-exponential decays (see Fig. S2 and S4†).

To further prove the appearance of triplet [DBP] in 4CzPN–*n*DBP, transient absorption spectroscopy (TAS) measurements were conducted. By selectively exciting the [4CzPN] unit with a 410 nm laser, characteristic absorption of ^3^[DBP] is detected, while the ground-state [DBP] is significantly bleached (Fig. S3†). The TAS-measured excited-state lifetimes of 4CzPN–*n*DBP are all above 200 μs, much longer than that of 4CzPN (Table 1). These results are consistent with the occurrence of an intramolecular triplet energy transfer process. Moreover, phosphorescence from ^3^[DBP] can be observed upon exciting 4CzPN–1DBP at 410 nm and 77 K in glassy 2-methyltetrahydrofuran (MeTHF) with a pulsed xenon lamp (Fig. S5†). This observation indicates that the triplet exciton can be transferred intramolecularly from [4CzPN] to [DBP] in the solid matrix at lowered temperature.

**Fig. 2:**
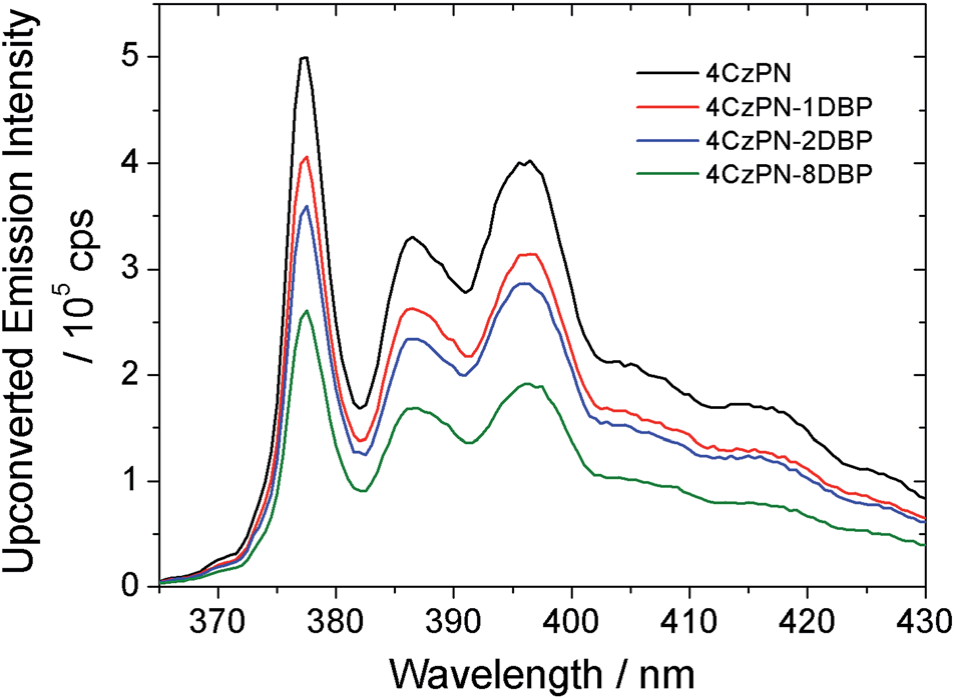
UC emission spectra of DBP (200 μM) in the presence of the studied photosensitizers (10 μM) in deaerated toluene.

We then tested the sensitizing abilities of 4CzPN–*n*DBP. Evident UC emissions were detected from deaerated toluene solutions of all three bichromophoric sensitizers in the presence of free DBP (Fig. 2). The UC quantum yields (*Φ*_UC_) of the systems sensitized by 4CzPN–1DBP, 4CzPN–2DBP and 4CzPN–8DBP (at 10 μM) were measured to be 3.7%, 2.7% and 1.7%, respectively. The slightly lowered *Φ*_UC_ of 4CzPN–1DBP compared to that of 4CzPN (4.4%) in solution suggests that, although the intramolecular triplet energy transfer to [DBP] effectively suppresses the reverse ISC and TADF, the triplet excitons received by tethered [DBP] are not efficiently passed on to the free emitters. We suspect that the excitons are likely lost through nonradiative decays. It was also noticed that as the number of linked [DBP] increased, the *Φ*_UC_ value further decreased.

**Fig. 3:**
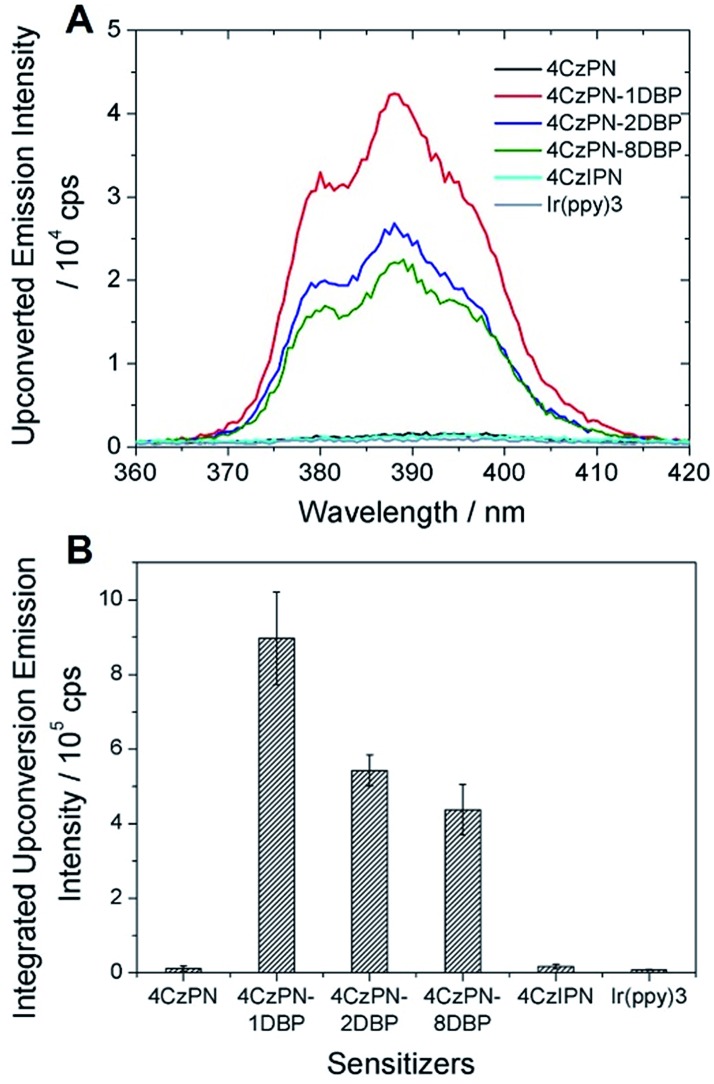
(A) UC emission spectra of the studied photosensitizers (5 μmol g^–1^) co-doped with DBP (50 μmol g^–1^) in PU films; (B) integrated UC emission intensities of DBP averaged from three parallel UC experiments.

In contrast to the solution-phase experiments, completely different results are obtained in the solid host. The DBP-appended sensitizers vastly outperform the non-functionalized CDCBs and accomplish much more efficient TTA UC in PU films. In these experiments, the non-coherent excitation light (∼7 mW cm^–2^) is provided by a 150 W Xe lamp, passing through a 450 ± 2.5 nm monochromator followed by a 400 nm long-pass filter to remove any high-energy photons. A short-pass filter (cut-off at ∼410 nm) was installed between the film and detector to remove the scattering light and residual fluorescence from the sensitizers. The UC emission spectra are shown in Fig. 3A and averaged data from three parallel experiments are summarized in Fig. 3B. All PU films containing sensitizer 4CzPN–*n*DBP co-doped with emitter DBP are observed to generate strong UC emissions. Evident UC signals can be detected from the PU films when the effective concentrations of 4CzPN–*n*DBP and emitter (free DBP) were merely 5 and 50 μmol g^–1^, respectively. In contrast, the UC emissions from 4CzPN and 4CzIPN-sensitized films are very weak under the same conditions. Among the three [DBP]-functionalized molecules, 4CzPN–1DBP exhibits the best performance. The average UC emission intensity from 4CzPN–1DBP-sensitized films is about 80 times that from 4CzPN-doped films. These results clearly demonstrate that covalently linking a [DBP] group to a CDCB molecule can significantly enhance the TTA UC efficiency in PU films, implying that the tethered [DBP] brings about a more efficient intermolecular TTET from the sensitizer to free DBP. The experiments also reveal that attaching more than one [DBP] unit to 4CzPN does not further improve the UC efficiency, but instead is harmful to the sensitizer performance (Fig. 3), likely due to the self-quenching effect among the [DBP] units in the same molecule. Such observations are also consistent with the previous observation that one [DBP] is sufficient to completely shut down TADF in 4CzPN (Table 1). Furthermore, the UC performance of the metal-free system in PU films was also compared to a metal–complex sensitizer, Ir(ppy)_3_. At the same sensitizer and emitter concentrations, a very weak UC signal is detected from films doped with the metal complex and DBP (Fig. 3).

**Fig. 4:**
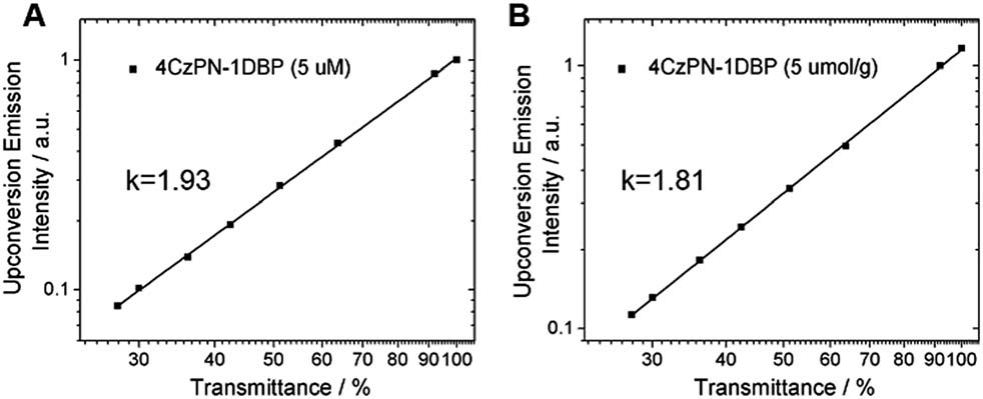
UC emission intensity of 4CzPN–1DBP (5 μM for A and 5 μmol g^–1^ for B) and DBP (300 μM for A and 50 μmol g^–1^ for B) plotted against the neutral density filter transmittance at 450 nm in (A) toluene and (B) PU films.

To prove that the observed UC emissions in toluene and the polymer matrix are truly generated *via* a TTA process, a quadratic dependence of the emission intensity on the excitation power is necessarily tested. For the sensitizer and emitter pair of 4CzPN–1DBP/DBP, the dependence of UC emission intensity on the incident light power was examined. As shown in Fig. 4, when the integrated intensity of the UC fluorescence from DBP is plotted against the transmittance percentage of the neutral density filter at 450 nm on a double-logarithmic scale,^21^ linear correlations with a slope close to 2 are observed for both solution and solid matrix, confirming the two-photon essence of the UC signals.^22^

**Fig. 5:**
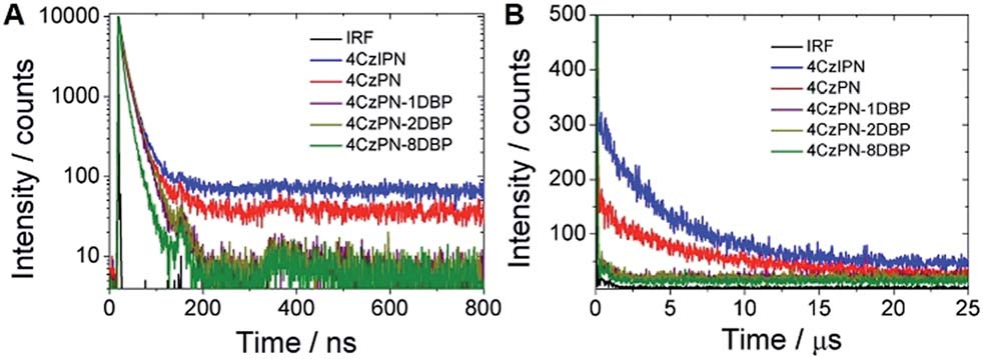
Prompt (A) and delayed (B) fluorescence decay profiles of five photosensitizers doped in PU films at 5 μmol g^–1^ (IRF refers to instrument response function).

To further confirm that intramolecular TTET has taken place with 4CzPN–*n*DBP dispersed in the polymer matrix, we then collected the time-resolved photoluminescence decay curves of the metal-free sensitizers dispersed in the PU films (Fig. 5). Long-lifetime TADF is clearly observable with 4CzPN and 4CzIPN, whereas the delayed fluorescence is nearly completely quenched in the case of 4CzPN–*n*DBP, supporting the conclusion that, for 4CzPN–*n*DBP in PU films, the triplet excitons generated by [4CzPN] are transferred to the tethered [DBP] unit highly efficiently.

**Fig. 6:**
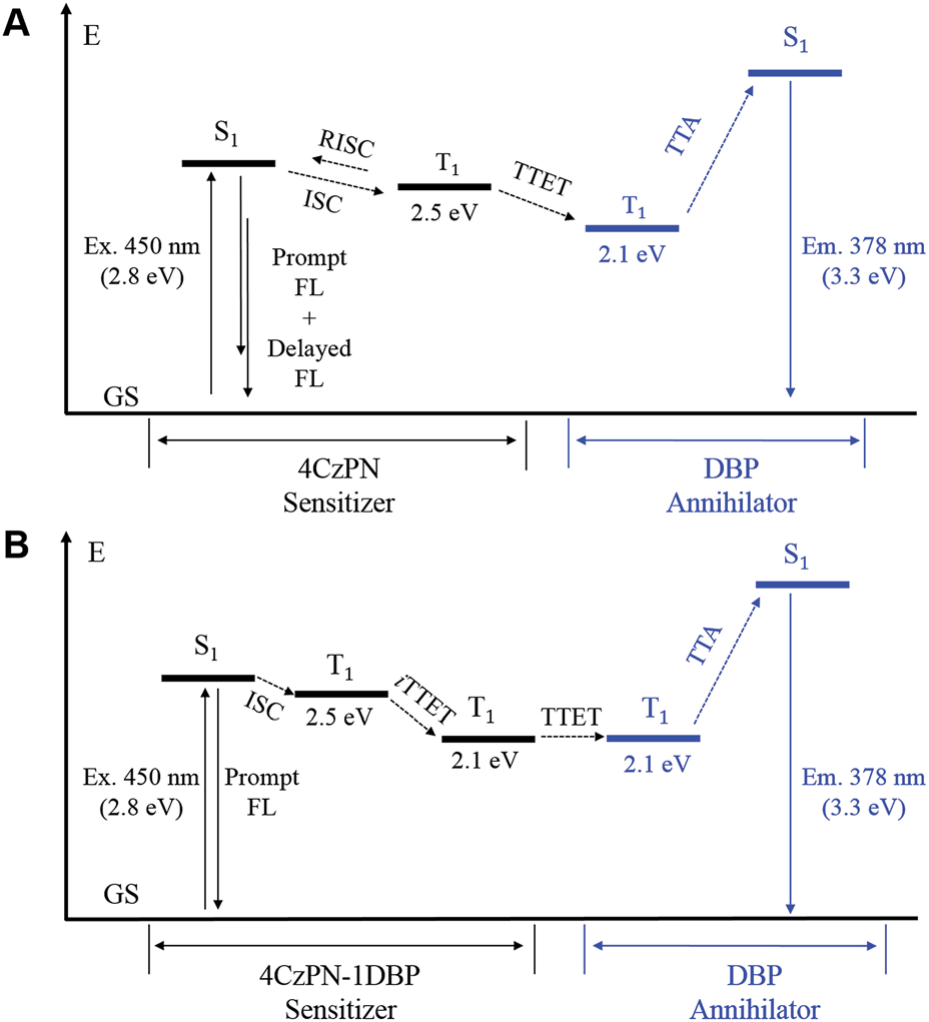
Energy diagrams of the important processes in TTA UC sensitized by 4CzPN (A) and 4CzPN–1DBP (B). The triplet energy levels were estimated based on the phosphorescence wavelengths at 77 K. FL: fluorescence; GS: ground state; RISC: reverse intersystem crossing; iTTET: intramolecular TTET; Ex.: excitation wavelength; Em.: emission maximum.

In essence, the above experimental results indicate that an effective intramolecular triplet energy transfer is established from [4CzPN] to [DBP] upon covalently ligating the two moieties together. Such an energy transfer pathway successfully suppresses the reverse ISC and TADF by competing for triplet excitons (Fig. 6). More importantly, this process plays a very critical role in achieving TTA UC in the solid matrix. Unlike in solution, the intermolecular TTET is significantly hampered in the diffusion-limited solid matrix. Consequently, the intermolecular TTET from 4CzPN to free DBP is nearly disabled, yielding the reverse ISC and TADF.

However, for 4CzPN–1DBP, the *in situ* generated 4CzPN–^3^[DBP] with a long lifetime allows the intermolecular TTET and subsequently TTA UC to proceed, rendering enhanced UC efficiency in PU films. An important note is that the nonradiative decay of 4CzPN–^3^[DBP] appears to be considerably slowed down in PU films compared to in solution.

## Conclusions

A series of heavy-atom-free triplet sensitizers were developed based on a TADF molecular scaffold (4CzPN), harnessing the small ΔE_ST_ induced ISC. Using low-power, non-coherent excitation light and DBP as the emitter, efficient TTA UC is realized with these new sensitizers in polymer films. Although the original TADF molecules are competent at sensitizing DBP in solution, under diffusion-limited conditions the intermolecular TTET is greatly hampered in competition with reverse ISC and TADF. By covalently ligating an energy acceptor (DBP) unit to 4CzPN (*i.e.*, an annihilator-appending strategy), triplet excitons are successfully removed from the singlet–triplet equilibrium within the 4CzPN moiety and become localized with the appended [DBP]. Namely, the intramolecular exciton transfer process supersedes the reverse ISC and subsequently enables TTA UC in association with the emitters dispersed in the PU films. To the best of our knowledge, this is the first time a heavy-atom-free photosensitizer has been developed and is fully functional for TTA UC in polymer films, promising more opportunities for realizing relevant applications in various fields.

## Supplementary Material

Supplementary information

Crystal structure data
