# Supplementary material for: Developing efficient heavy-atom-free photosensitizers applicable to TTA upconversion in polymer films
Source: Chem Sci. 2015 Nov 9;7(2):1233–7. doi: 10.1039/c5sc03245h (PMC5975838; doi:10.1039/c5sc03245h)
Supplement: Supplementary file 1 [file SC-007-C5SC03245H-s001.pdf]

Electronic Supplementary Material (ESI) for Chemical Science.
This journal is © The Royal Society of Chemistry 2015

ELECTRONIC SUPPORTING INFORMATION

Developing efficient heavy-atom-free photosensitizers applicable to TTA upconversion in polymer films

Jiang Peng, Xinyan Guo, Xinpeng Jiang, Dahui Zhao*, Yuguo Ma*

Beijing National Laboratory for Molecular Sciences, Center for Soft Matter Science and Engineering, Key Lab of Polymer Chemistry & Physics of Ministry of Education, College of Chemistry, Peking University, Beijing 100871, China

ygma@pku.edu.cn (Yuguo Ma)
dhzhao@pku.edu.cn (Dahui Zhao)

Contents

Synthetic methods and characterization data……………….............…………….......S2

Photophysical measurements……………………………………………………...….S9

References……………………………………………………………………………..S14

^{1}H and ^{13}C NMR spectra of the target molecules……….……….......................….......S15

X-ray structural analysis of 3CzFPN…………………………………………………..S18

Synthetic methods and characterization data

General Methods

All reagents were commercial products and unless otherwise indicated they were used directly without any further purification. Tetrahydrofuran (THF), toluene, chloroform and dichloromethane (DCM) were purchased from Beijing Tong Guang Fine Chemicals Company. THF was freshly distilled over sodium under nitrogen atmosphere prior to use. DCM was stirred overnight with CaH_2 and then distilled. Chloroform was used without further purification. ^{1}H NMR and ^{13}C NMR spectra were collected with Bruker ARX400 (400 MHz) or Bruker Avance III (500 MHz) instruments, using $CDCl_3$ as solvent. ^{19}F NMR spectrum was collected with a Varian Mercury-300 (300 MHz) instrument. Chemical shifts are reported in parts per million (ppm) and coupling constants are reported in Hertz (Hz). ^{1}H and ^{13}C NMR chemical shifts were referenced to the residual solvent peak (7.26 ppm for ^{1}H and 77.0 ppm for ^{13}C). ^{19}F NMR chemical shifts were referenced versus a CF_3CO_2H external standard (0 ppm). Single crystal X-ray diffraction data was collected with a NONIUS Kappa CCD diffractometer for 3CzFPN, with graphite monochromator and Mo Kα radiation [$\lambda(Mo_{K\alpha})$ = 0.71073 Å]. Structures were solved by direct methods with SHELXS-97 and refined against F^2 with SHELXS-97. Electro-spray ionization (ESI) mass spectrometry was performed using a Bruker Apex IV FTMS instrument. Matrix-assisted laser desorption ionization (MALDI) mass spectrometry was performed using AB Sciex MALDI-TOF/TOF Mass Spectrometer.

Synthetic procedures

4CzPN and 4CzIPN were synthesized according to the literature[1].

General strategy for the syntheses of DBP attached 4CzPN

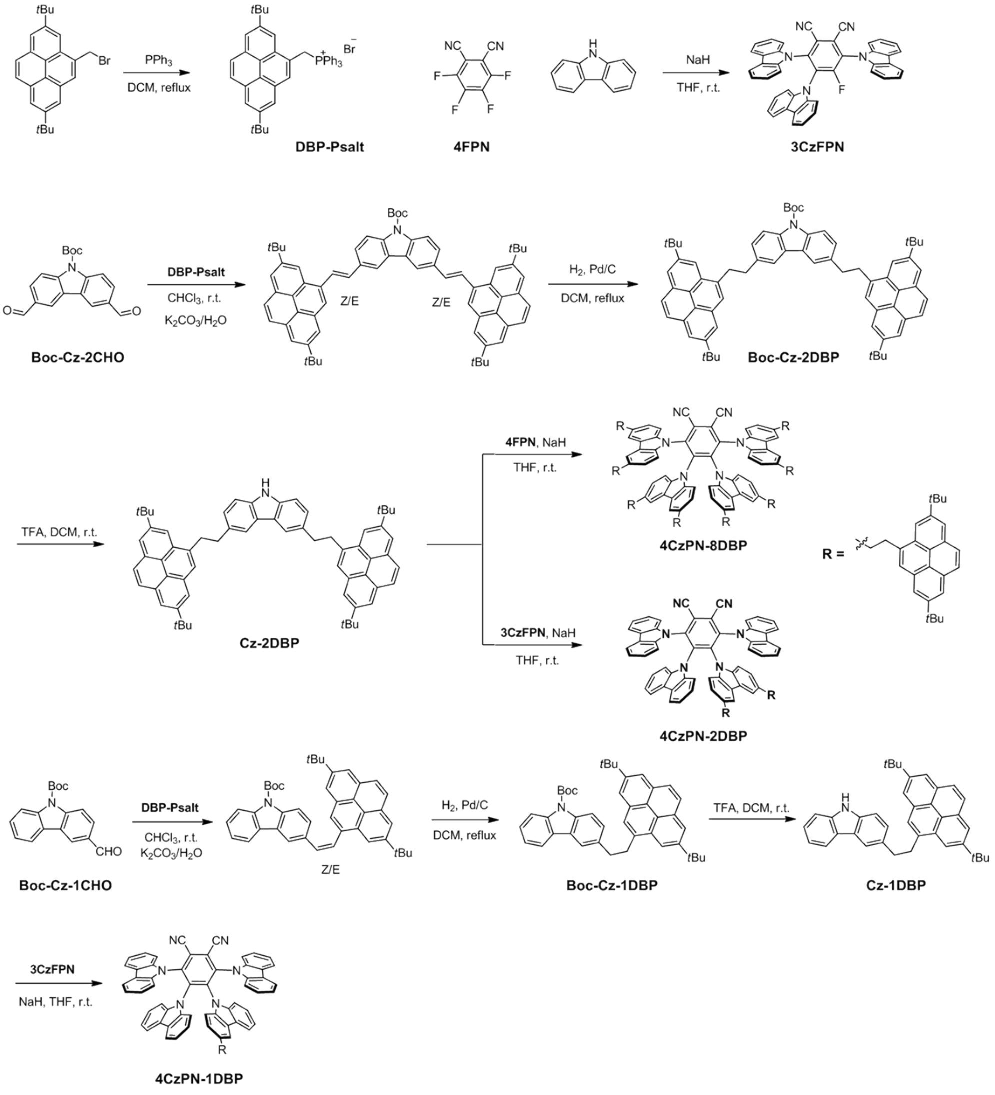

Synthetic methods and characterization data

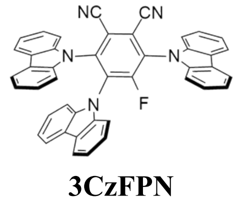

3CzFPN

NaH (144 mg, 6 mmol) was added to a stirred solution of carbazole (1000 mg, 6 mmol) in dry THF (15 ml) at room temperature. After stirred for 30 min, the suspension was slowly added to the solution which was prepared by dissolving tetrafluorophthalonitrile (400 mg, 2.00 mmol) in THF (10 mL). The reaction mixture was stirred at room temperature overnight. The reaction was quenched with water (1 ml). The mixture was diluted with 100 mL DCM and washed with water, aqueous ammonium chloride, and brine in sequence. The organic layer was separated and dried over anhydrous Na_2SO_4, filtered and concentrated *in vacuo*.

The residue was subjected to column chromatography (silica gel, DCM/PE, 2/3, v/v) to afford **3CzFPN** (65 mg, 5%) as a yellow solid. The substitution position of fluorine atom was identified by X-ray structural analysis of **3CzFPN** single crystal (see below). ^{1}H NMR (400 MHz, $CDCl_3$): δ 8.19 (d, 2H, J = 7.6 Hz), 7.73 (d, 2H, J = 7.6 Hz), 7.67 (d, 2H, J = 7.6 Hz), 7.58 (t, 2H, J = 7.6 Hz), 7.45 (d, 2H, J = 7.2 Hz), 7.41 (m, 2H), 7.11 (m, 8H), 7.00 (t, 2H, J = 7.6 Hz), 6.89 (d, 2H, J = 7.2 Hz). ^{19}F NMR (282 MHz, $CDCl_3$): -25.2. ESI-HRMS (m/z): $[M+H]^+$ calculated for $C_{44}H_{25}FN_5^+$, 642.2089; found, 642.2078; $[M+NH_4]^+$ calculated for $C_{44}H_{28}FN_6^+$, 659.2354; found, 659.2355.

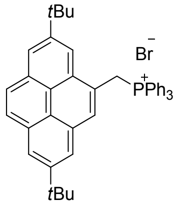

DBP-Psalt

Triphenylphosphine (232 mg, 0.88 mmol) was added in a solution of **4-(bromomethyl)-2,7-di-*tert*-butylpyrene** (60 mg, 0.15 mmol) in dichloromethane (5 mL). The mixture was stirred at reflux overnight and then cooled to room temperature. After removal of dichloromethane *in vacuo*, the residue was subjected to column chromatography (silica gel, DCM/CH_3OH, 15/1, v/v) to afford **DBP-Psalt** (63 mg, 64%) as a white solid. ESI-HRMS (m/z): $[M-Br^-]^+$ calcd for $C_{43}H_{42}P^+$: 589.3019; found 589.3001.

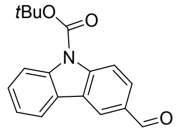

Boc-Cz-1CHO

Boc-Cz-1CHO was synthesized according to the literature[2]. ^{1}H NMR (400 MHz, $CDCl_3$): δ 10.11 (s, 1H), 8.47 (s, 1H), 8.45 (d, 1H, J = 8.7 Hz), 8.29 (d, 1H, J = 8.4 Hz), 8.03 (d, 1H, J = 7.5 Hz), 7.98 (dd, 1H, J = 8.7 Hz, 1.4 Hz), 7.54-7.50 (m, 1H), 7.41 (t, 1H, J = 7.5 Hz), 1.78 (s, 9H).

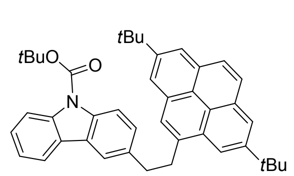

Boc-Cz-1DBP

Aqueous potassium carbonate (2.1 g, 3 M) was added dropwisely to a solution of **Boc-Cz-1CHO** (300 mg, 1.02 mmol) and **DBP-Psalt** (816 mg, 1.22 mmol) in chloroform (15 mL). The mixture was stirred at room temperature for 10 h. The organic layer was separated and then washed with water and brine, dried over anhydrous Na_2SO_4, filtered and concentrated *in vacuo*. The solid residue was subjected to column chromatography (silica gel, DCM/petroleum ether (PE), 1/3, v/v) to get the corresponding olefin as a white solid with blue fluorescence. Without further purification, the solid was added to a suspension of Pd/C

(12 mg) in THF (70 mL). The mixture was stirred on 2.5 atm hydrogen at 55 °C for 48 h and then filtered off from insoluble Pd/C. After removal of the solvent *in vacuo*, the residue was subjected to column chromatography (silica gel, DCM/PE, 1/3, v/v) to afford **Boc-Cz-1DBP** (330 mg, 54%) as a white solid. ^{1}H NMR (400 MHz, $CDCl_3$): δ 8.47 (s, 1H), 8.32 (d, 1H, J = 8.4 Hz), 8.26 (d, 1H, J = 8.4 Hz), 8.21 (s, 1H), 8.15-8.13 (m, 2H), 8.05 (d, 1H, J = 8.8 Hz), 8.02 (d, 1H, J = 8.8 Hz), 7.96-7.95 (m, 3H), 7.49-7.44 (m, 2H), 7.37-7.33 (m, 1H), 3.64-3.68 (m, 2H), 3.42-3.38 (m, 2H), 1.77 (s, 9H), 1.61 (s, 9H), 1.57 (s, 9H). ^{13}C NMR (100 MHz, $CDCl_3$): δ 151.2, 148.7, 148.2, 138.9, 137.2, 137.1, 136.9, 131.3, 130.7, 130.0, 127.7, 127.6, 127.3, 127.0, 126.5, 126.1, 125.8, 123.3, 123.0, 122.2, 122.0, 121.8, 121.6, 119.5, 119.2, 118.3, 116.3, 83.8, 36.8, 36.5, 35.4, 35.2, 32.1, 32.0, 28.4. ESI-HRMS (m/z): $[M+H]^+$ calculated for $C_{43}H_{46}NO_2^+$, 608.3523; found, 608.3529.

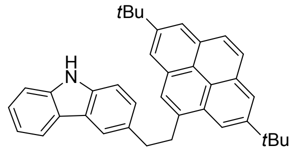

Cz-1DBP

Boc-Cz-1DBP (50 mg, 0.082 mmol) was dissolved in a mixture of DCM (10 mL) and trifluoroacetic acid (TFA, 3mL). The mixture was stirred for 2 h at room temperature. The reaction mixture was washed with water, sodium bicarbonate, and brine in sequence. The organic layer was separated and dried over anhydrous Na_2SO_4, filtered and concentrated *in vacuo*. The solid residue was subjected to column chromatography (silica gel, DCM/PE, 1/2, v/v) to afford **Cz-1DBP** (31 mg, 74%) as a white solid. ^{1}H NMR (400 MHz, $CDCl_3$): δ 8.50 (d, 1H, J = 1.2 Hz), 8.21 (d, 1H, J = 1.2 Hz), 8.15 (s, 2H), 8.07-8.00 (m, 4H), 7.96 (s, 1H), 7.81 (s, 1H), 7.41-7.19 (m, 5H), 3.68-3.64 (m, 2H), 3.42-3.37 (m, 2H), 1.61 (s, 9H), 1.57 (s, 9H). ^{13}C NMR (100 MHz, $CDCl_3$): δ 148.7, 148.3, 139.9, 138.2, 137.2, 133.5, 131.4, 130.8, 130.7, 130.1, 127.7, 127.4, 126.7, 126.5, 125.8, 123.7, 123.4, 123.3, 122.2, 122.0, 121.8, 121.6, 120.3, 119.8, 119.4, 118.5, 37.1, 37.0, 35.5, 35.3, 32.1, 32.0. ESI-HRMS (m/z): $[M+H]^+$ calculated for $C_{38}H_{38}N^+$, 508.2999; found, 508.3001.

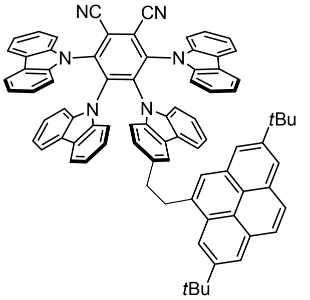

4CzPN-1DBP

4CzPN-1DBP was synthesized according to the modified procedure.[1] NaH (15 mg, 0.63 mmol) was added to a stirred solution of **Cz-1DBP** (31 mg, 0.06 mmol) in dry THF (5 ml) at room temperature. After stirring for 30 min, **3CzFPN** (20 mg, 0.03 mmol) was added. The reaction mixture was stirred at room temperature for 5 h. The reaction was quenched with water (1 ml). The mixture was diluted with 20 mL DCM and washed with water, aqueous

ammonium chloride, and brine in sequence. The organic layer was separated and dried over anhydrous Na_2SO_4, filtered and concentrated *in vacuo*. The residue was subjected to column chromatography (silica gel, DCM/PE, 1/1, v/v) to afford **4CzPN-1DBP** (6.2 mg, 18%) as a yellow solid. ^{1}H NMR (500 MHz, $CDCl_3$): δ 8.35 (d, 1H, J = 1.6 Hz), 8.21 (d, 1H, J = 1.6 Hz), 8.15 (d, 1H, J = 1.6 Hz), 8.09 (d, 1H, J = 1.6 Hz), 8.04 (d, 1H, J = 9.0 Hz), 8.02 (d, 1H, J = 9.0 Hz), 7.82 (s, 1H), 7.75-7.70 (m, 4H), 7.27-7.24 (m, 6H), 7.18-7.01 (m, 10H), 6.93-6.74 (m, 7H), 6.62-6.55 (m, 4H), 3.34-3.30 (m, 2H), 3.03-3.00 (m, 2H), 1.62 (s, 9H), 1.56 (s, 9H). ESI-HRMS (m/z): $[M+NH_4]^+$ calculated for $C_{82}H_{64}N_7^+$, 1146.5218; found, 1146.5209.

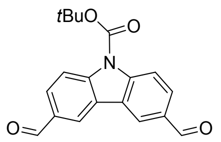

Boc-Cz-2CHO

Boc-Cz-2CHO was synthesized according to the literature[2].

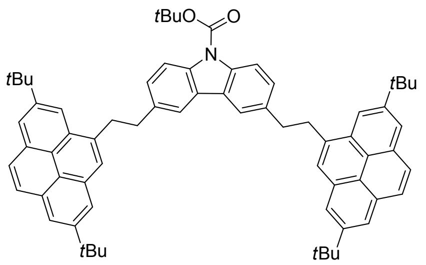

Boc-Cz-2DBP

Aqueous potassium carbonate (2.6 g, 3 M) was added dropwisely to a solution of **Boc-Cz-2CHO** (200 mg, 0.62 mmol) and **DBP-Psalt** (1.0 g, 1.49 mmol) in chloroform (15 mL). The mixture was stirred at room temperature for 13 h. The organic layer was separated and then washed with water and brine, dried over anhydrous Na_2SO_4, filtered and concentrated *in vacuo*. The solid residue was subjected to column chromatography (silica gel, DCM/PE, 1/4, v/v) to get the Z/E isomers of olefin as a white solid with blue fluorescence. Without further purification, the solid was added to a suspension of Pd/C (15 mg) in THF (70 mL). The mixture was stirred on 2.5 atm hydrogen at 55 °C for 48 h and then filtered off from insoluble Pd/C. After removal of the solvent *in vacuo*, the residue was subjected to column chromatography (silica gel, DCM/PE, 1/3, v/v) to afford **Boc-Cz-2DBP** (240 mg, 41%) as a white solid. ^{1}H NMR (400 MHz, $CDCl_3$): δ 8.47 (s, 2H), 8.27 (d, 2H, J = 8.4 Hz), 8.21 (s, 2H), 8.15 (s. 4H), 8.05 (d, 2H, J = 9.6 Hz), 8.02 (d, 2H, 9.6 Hz), 7.95 (s, 2H), 7.92 (s, 2H), 7.46 (d, 2H, J = 8.4 Hz), 3.69-3.65 (m, 4H), 3.43-3.39 (m, 4H), 1.78 (s, 9H), 1.608 (s, 9H), 1.61 (s, 9H), 1.57 (s, 9H), 1.57 (s, 9H). ^{13}C NMR (100 MHz, $CDCl_3$): δ 151.2, 148.7, 148.2, 137.4, 137.1, 136.9, 131.3, 130.7, 130.0, 127.6, 127.6, 127.3, 126.5, 126.1, 123.3, 122.2, 122.0, 121.8, 121.6, 119.2, 118.3, 116.3, 83.7, 36.8, 36.5, 35.4, 35.2, 32.1, 32.0, 28.5, 26.9. ESI-HRMS (m/z): $[M+H]^+$ calculated for $C_{69}H_{74}NO_2^+$, 948.5714; found, 948.5695.

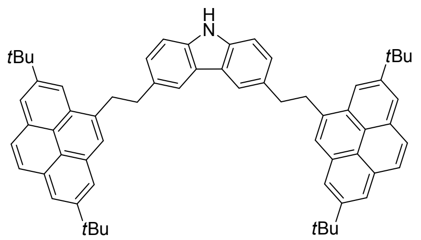

Cz-2DBP

Boc-Cz-2DBP (210 mg, 0.22 mmol) was dissolved in a mixture of DCM (15 mL) and TFA (5 mL) and stirred for 2 h at room temperature. The reaction mixture was washed with water, sodium bicarbonate, and brine in sequence. The organic layer was separated and dried over anhydrous Na_2SO_4, filtered and concentrated *in vacuo*. The solid residue was subjected to column chromatography (silica gel, DCM/PE, 2/5, v/v) to afford **Cz-2DBP** (149 mg, 80%) as a white solid. ^1H NMR (400 MHz, $CDCl_3$): δ 8.51 (d, 2H, J = 1.6 Hz), 8.22 (d, 2H, J = 1.6 Hz), 8.15 (s, 4H), 8.09 (s, 2H), 8.05 (d, 2H, J = 8.8 Hz), 8.03 (d, 2H, J = 8.8 Hz), 8.00 (s, 1H), 7.98 (s, 2H), 7.42 (d, 2H, J = 8.8 Hz), 7.40 (d, 2H, J = 8.8 Hz), 3.71-3.67 (m, 4H), 3.45-3.41 (m, 4H), 1.61 (s, 18H), 1.58 (s, 18H). ^{13}C NMR (100 MHz, $CDCl_3$): δ 148.7, 148.3, 138.6, 137.2, 133.4, 131.3, 130.8, 130.7, 130.1, 127.7, 127.3, 126.6, 126.4, 123.6, 123.3, 122.2, 122.0, 121.8, 121.5, 119.8, 118.5, 110.6, 37.1, 36.9, 35.5, 35.2, 32.1, 32.0. ESI-HRMS (m/z): $[M+Na]^+$ calculated for $C_{64}H_{65}NNa^+$, 870.5009; found, 870.5023; $[M+K]^+$ calculated for $C_{64}H_{65}KN^+$, 886.4749; found, 886.4763.

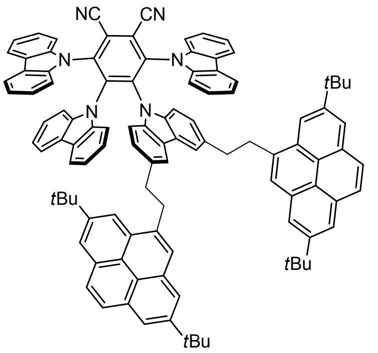

4CzPN-2DBP

4CzPN-2DBP was synthesized according to the modified procedure. NaH (5 mg, 0.21 mmol) was added to a stirred solution of **Cz-2DBP** (17 mg, 0.02 mmol) in dry THF (5 ml) at room temperature. After stirring for 30 min, **3CzFPN** (6.4 mg, 0.01 mmol) was added. The reaction mixture was stirred at room temperature for 8 h. The reaction was quenched with water (1 ml). The mixture was diluted with 20 mL DCM and washed with water, aqueous ammonium chloride, and brine in sequence. The organic layer was separated and dried over anhydrous Na_2SO_4, filtered and concentrated *in vacuo*. The residue was subjected to column chromatography (silica gel, DCM/PE, 1/1, v/v) to afford **4CzPN-2DBP** (4.8 mg, 33%) as a yellow solid. ^1H NMR (500 MHz, $CDCl_3$): δ 8.36 (d, 2H, J = 1.2 Hz), 8.21 (d, 2H, J = 1.2 Hz), 8.15 (d, 2H, J = 1.5 Hz), 8.11 (d, 2H, J = 1.5 Hz), 8.04 (d, 2H, J = 9.0 Hz), 8.02 (d, 2H, J = 9.0 Hz), 7.85 (s, 2H), 7.75 (d, 2H, J = 8.0 Hz), 7.72-7.68 (m, 2H), 7.27-7.21 (m, 8H), 7.14-7.06 (m, 8H), 6.93 (d, 2H, J = 8.5 Hz), 6.83 (d, 2H, J = 8.5 Hz), 6.77-6.74 (m, 2H),

6.62-6.57 (m, 4H), 3.36-3.33 (m, 4H), 3.05-3.02 (m, 4H), 1.61 (s, 18H), 1.56 (s, 18H). ESI-HRMS (m/z): $[M+NH_4]^+$ calculated for $C_{108}H_{92}N_7^+$, 1486.7409; found, 1486.7438.

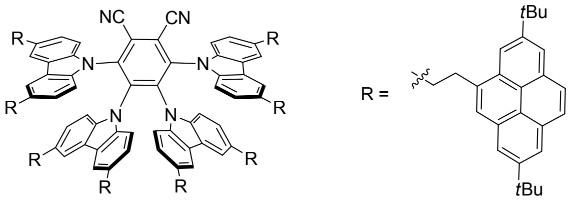

4CzPN-8DBP

4CzPN-8DBP was synthesized according to the modified procedure.[1] NaH (12 mg, 0.50 mmol) was added to a stirred solution of **Cz-2DBP** (41 mg, 0.05 mmol) in dry THF (5 ml) at room temperature. After stirring for 30 min, **4FPN** (1.6 mg, 0.01 mmol) was added. The reaction mixture was stirred at room temperature for 18 h. The reaction was quenched with water (1 ml). The mixture was diluted with 20 mL DCM and washed with water, aqueous ammonium chloride, and brine in sequence. The organic layer was separated and dried over anhydrous Na_2SO_4, filtered and concentrated *in vacuo*. The residue was subjected to column chromatography (silica gel, DCM/PE, 1/3, v/v) to afford **4CzPN-8DBP** (8.2 mg, 29%) as a yellow solid. ^1H NMR (500 MHz, $CDCl_3$): δ 8.46 (s, 4H), 8.34 (s, 4H), 8.14 (s, 4H), 8.09 (d, 4H, J = 1.4 Hz), 8.04 (s, 4H), 8.01 (d, 4H, J = 1.3 Hz), 7.99-7.94 (m, 12H), 7.88 (d, 4H, J = 9.2 Hz), 7.86 (d, 4H, J = 9.2 Hz), 7.84 (s, 4H), 7.80 (s, 8H), 7.70 (s, 4H), 7.44 (s, 4H), 7.32 (d, 4H, J = 7.7 Hz), 7.16 (d, 4H, J = 7.7 Hz), 7.11 (d, 4H, J = 8.4 Hz), 6.81 (d, 4H, J = 8.4 Hz), 3.66-3.57 (m, 8H), 3.44-3.41 (m, 8H), 3.35-3.25 (m, 8H), 3.07-3.04 (m, 8H), 1.52 (s, 36H), 1.48 (s, 36H), 1.38 (s, 72H). MALDI-TOF-MS (m/z): $[M+Na]^+$ calculated for $C_{264}H_{256}N_6Na^+$, 3533.01; found, 3533.35; $[M+K]^+$ calculated for $C_{264}H_{256}KN_6^+$, 3548.98; found, 3549.35.

Photophysical measurements

Photophysical measurements in solution

Steady state spectroscopy. All the measurements were carried out using freshly distilled DCM or toluene as solvents. The deaerated toluene solutions were prepared by three cycles of freeze-pump-thaw and re-filled with nitrogen. UV-vis absorption spectra were recorded using a Hitachi U-4100 spectrophotometer with a 1-cm quartz cell. Photoluminescence spectra were recorded on a Horiba Jobin Yvon FluoroMax-4P spectrofluorometer with right-angle geometry, using 1-cm quartz cuvettes. The excitation wavelength in the quantum yield measurements of the sensitizers was 380 nm and $Ru(bpy)_3Cl_2$ (Φ_p = 0.04 in aerated water) was used as quantum yield standard.[3] The phosphorescence spectra at 77 K were recorded on a Horiba Jobin Yvon FluoroMax-4P spectrofluorometer with right-angle geometry using quartz tube in 2-methyltetrahydrofuran (MTHF) matrix at liquid-nitrogen temperature.

The plots in Figure 3 was fitted according to the Stern-Volmer equation: $I_0/I=1+K_{SV}[Q]$. I_0 is the photoluminescence intensity in the absence of the quencher while I is the intensity in the presence of the quencher; [Q] is the molar concentration of the quencher, and K_{SV} is the Stern-Volmer quenching constant.

Lifetime measurement. Photoluminescence decay curves were collected on a Horiba Jobin Yvon FluoroMax-4P spectrofluorometer. Samples for delayed fluorescence lifetime measurements were prepared in freshly distilled toluene. The solutions were degassed by three cycles of freeze-pump-thaw and re-filled with nitrogen. Lifetimes for prompt fluorescence or delayed fluorescence were measured with time correlated single photon counting (TCSPC) using a FluoroMax-4P spectrofluorometer with NanoLED of 369 nm as the excitation source. Full width at half-maximum (FWHM) of the instrument response function (IRF) obtained by an aqueous solution of Ludox® colloidal silica was typically ~1 ns in our system. The prompt fluorescence lifetimes were fitted with single exponential or two exponential decay with deconvolution and the delayed fluorescence lifetimes were fitted without deconvolution. The exponential fits were performed using Horiba Jobin Yvon DAS6 software.

Nanosecond Transient Absorption. Nanosecond transient absorption measurements were performed on a LP-920 laser flash photolysis setup (Edinburgh). Excitation at 410 nm with a computer-controlled Nd:YAG laser/OPO system from Opotek (Vibrant 355 II) operating at 10 Hz was directed to the samples with O.D. of ~0.1 at the excitation wavelength. Both the fluorescence lifetime and transient absorption spectra of 4CzPN-8DBP were two-exponential decay (Figure S2C and Figure S4D).

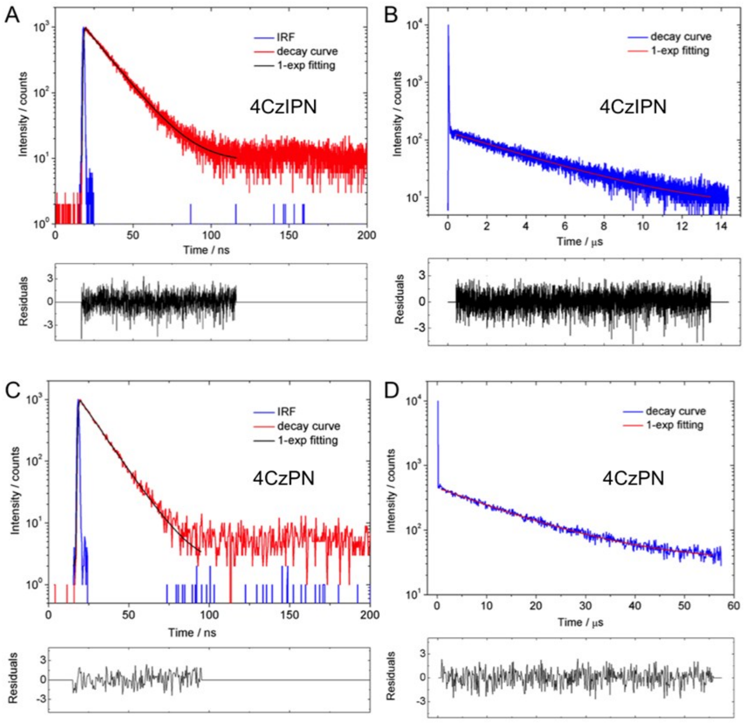

Figure S1 Prompt (A, C) and delayed (B, D) fluorescence decay curves of 4CzIPN and 4CzPN in deaerated toluene and the corresponding exponential fitting. IRF refers to instrument response function.

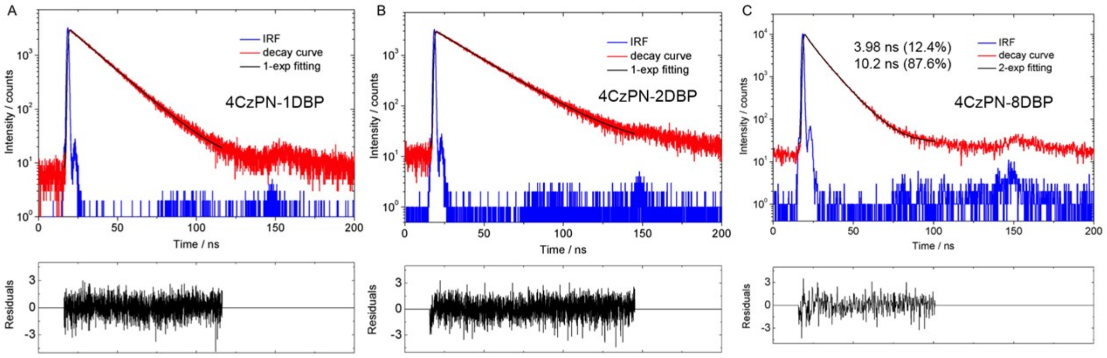

Figure S2 Prompt fluorescence decay curves of 4CzPN derivatives in deaerated toluene and the corresponding exponential fitting. IRF refers to instrument response function.

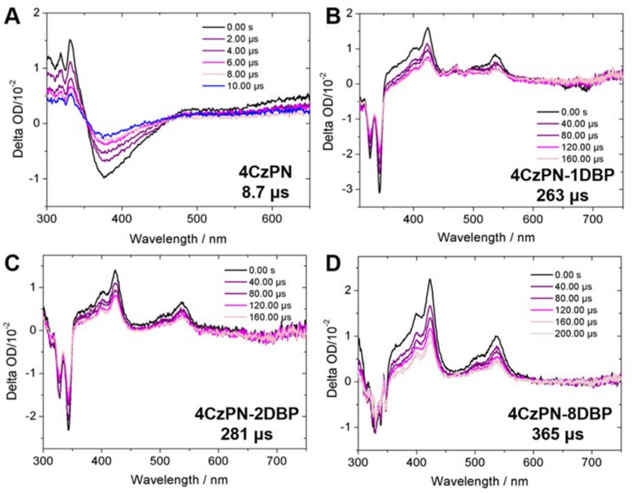

Figure S3 Transient absorption difference spectra of (A) 4CzPN, (B) 4CzPN-1DBP, (C) 4CzPN-2DBP and (D) 4CzPN-8DBP at 10 μM in deaerated toluene. The samples were excited at 410 nm with a pulsed laser (~10 mJ/pulse, FWHM = 8 ns) at 20 °C.

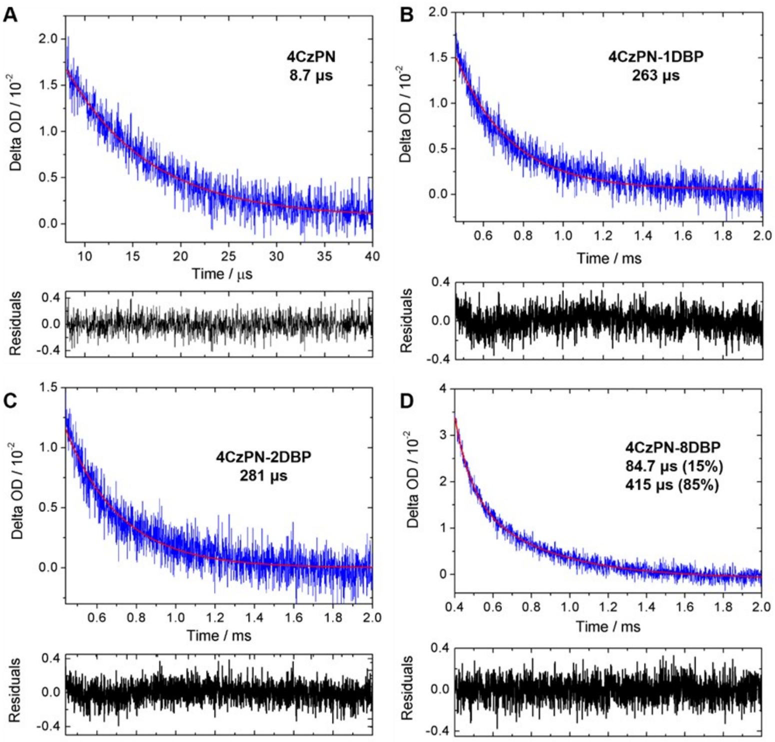

Figure S4 Transient absorption decays of 4CzPN and 4CzPN-nDBP with exponential fits to the 375 nm transient (for 4CzPN) or 430 nm transient (for 4CzPN-nDBP) with residuals presented in the bottom.

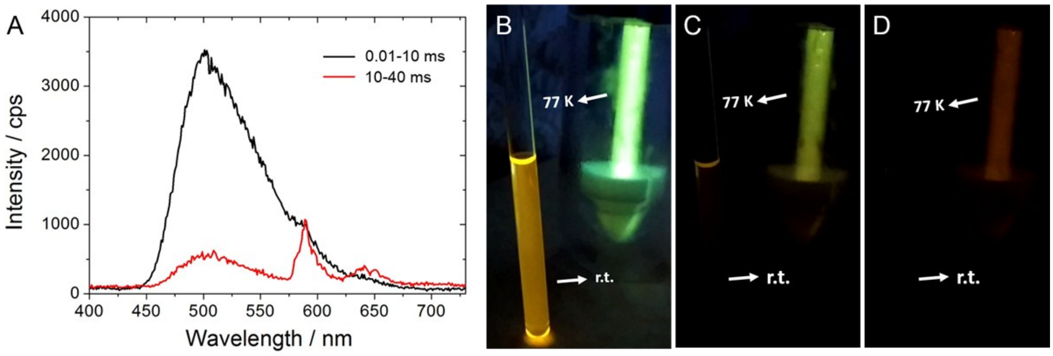

Figure S5 (A) Phosphorescence spectra of 4CzPN-1DBP (5×10^{-5} M in MTHF at 77 K, excited at 370 nm by a pulsed Xe lamp) collected with a certain delay time; (B, C, D) Screenshots of a video of 4CzPN-1DBP in MTHF at room temperature (left) and at 77 K (right) under a 365 nm UV lamp: (B) the lamp was on; (C, D) shortly (~0.2 s) after the lamp was turned off (recorded in sequence).

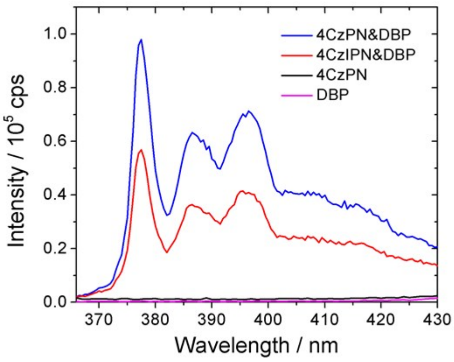

Figure S6 4CzPN (10 μM) or 4CzIPN (10 μM) sensitized TTA UC spectra of DBP (100 μM) in deaerated toluene (excited at 450 nm with a Xe lamp).

Photophysical measurements in polyurethane (PU) films

The experimental conditions of steady state spectroscopy and lifetime measurements for the PU films were the same as described above for measurements in solution.

Preparation of PU films. The procedure was based on modified method from literature.[4] Polycaprolactone diol (860 mg, ~1.72 mmol) and methylene bis(4-cyclohexyl isocyanate) (500 mg, 1.9 mmol) were mixed with tetramethylethylenediamine (TMEDA) in THF (2.5 mL). After thorough mixing and curing for 12 h at room temperature, the samples were subsequently placed in a conventional oven at 100 °C for 10 h, and PU was obtained as transparent and elastic polymer.

PU (1.0 g) polymer was dissolved in THF (5 mL). The studied compounds (the sensitizer and the annihilator) were dissolved in THF solution of PU. After thoroughly mixing, the solution was drop-casted on a quartz slide, and the transparent uniform film was obtained. The films were placed in a vacuum oven at 40 °C for 12 h to remove any residual THF. Unless otherwise noted, the doping concentrations were 5 μmol / 1 g PU for the sensitizer and 50 μmol / 1 g PU for the annihilator.

TTA UC measurements. The upconversion spectra were recorded on a Horiba Jobin Yvon FluoroMax-4P spectrofluorometer with incident angle of 30° for the film and a short pass filter was put on the detection light path to reduce the scattered light. The transmittance of the

short pass filter and the UC emission spectra of DBP after passing the short pass filter were presented in Figure S7.

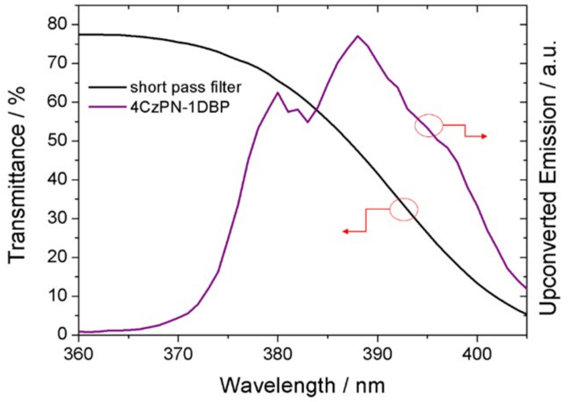

Figure S7 Transmittance of the short pass filter (black curve) and DBP upconverted emission profile sensitized by 4CzPN-1DBP measured with the short pass filter in the detection light path (purple curve).

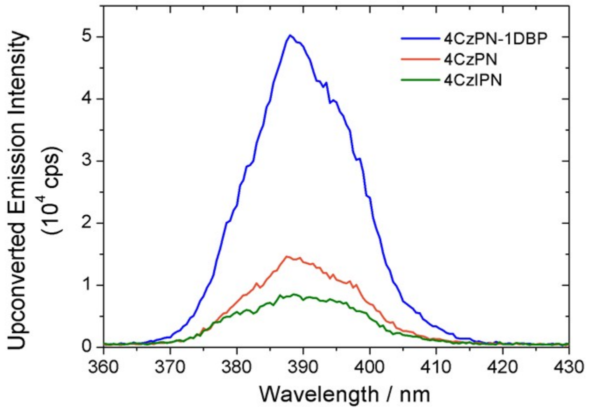

Figure S8 Upconversion emission profiles of DBP in PU films sensitized by 4CzIPN, 4CzPN or 4CzPN-1DBP. The doping concentrations were 5 μmol/g for sensitizers and 150 μmol/g for DBP. The spectra were recorded with the short pass filter (excited at 450 ± 2.5 nm using a Xe lamp with a 400 nm long pass filter).

References

1. Uoyama, H.; Goushi, K.; Shizu, K.; Nomura, H.; Adachi, C. *Nature* **2012**, *492*, 234-238.
2. (a) Patrick, D.; Boykin, D.; Wilson, W.; Tanious, F.; Spychala, J.; Bender, B.; Hall, J.; Dykstra, C.; Ohemeng, K.; Tidwell, R. *Eur. J. Med. Chem.* **1997**, *32*, 781-793; (b) Moorthy, J. N.; Mandal, S.; Mukhopadhyay, A.; Samanta, S. *J. Am. Chem. Soc*. **2013**, *135*, 6872-6884.
3. Suzuki, K.; Kobayashi, A.; Kaneko, S.; Takehira, K.; Yoshihara, T.; Ishida, H.; Shiina, Y.; Oishi, S.; Tobita, S. *Phys. Chem. Chem. Phys*. **2009**, *11*, 9850-9860.
4. Kim, J.-H.; Deng, F.; Castellano, F. N.; Kim, J.-H. *Chem. Mater*. **2012**, *24*, 2250-2252.

^{1}H NMR and ^{13}C NMR spectra of the target molecules

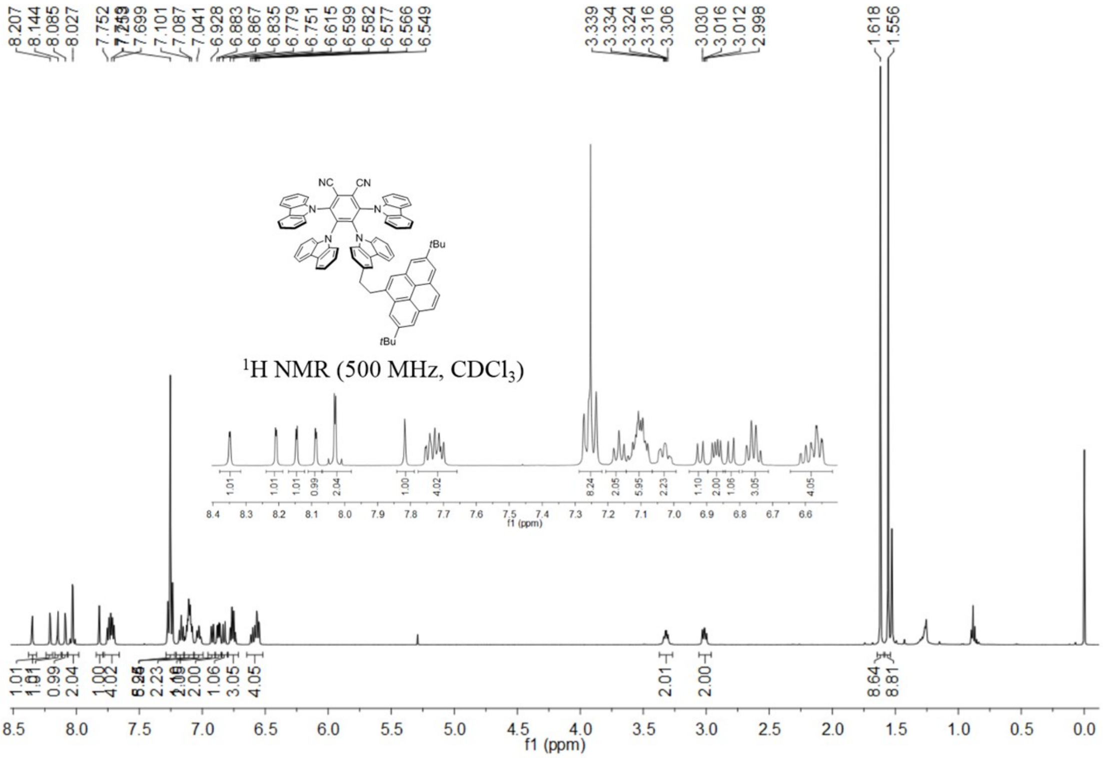

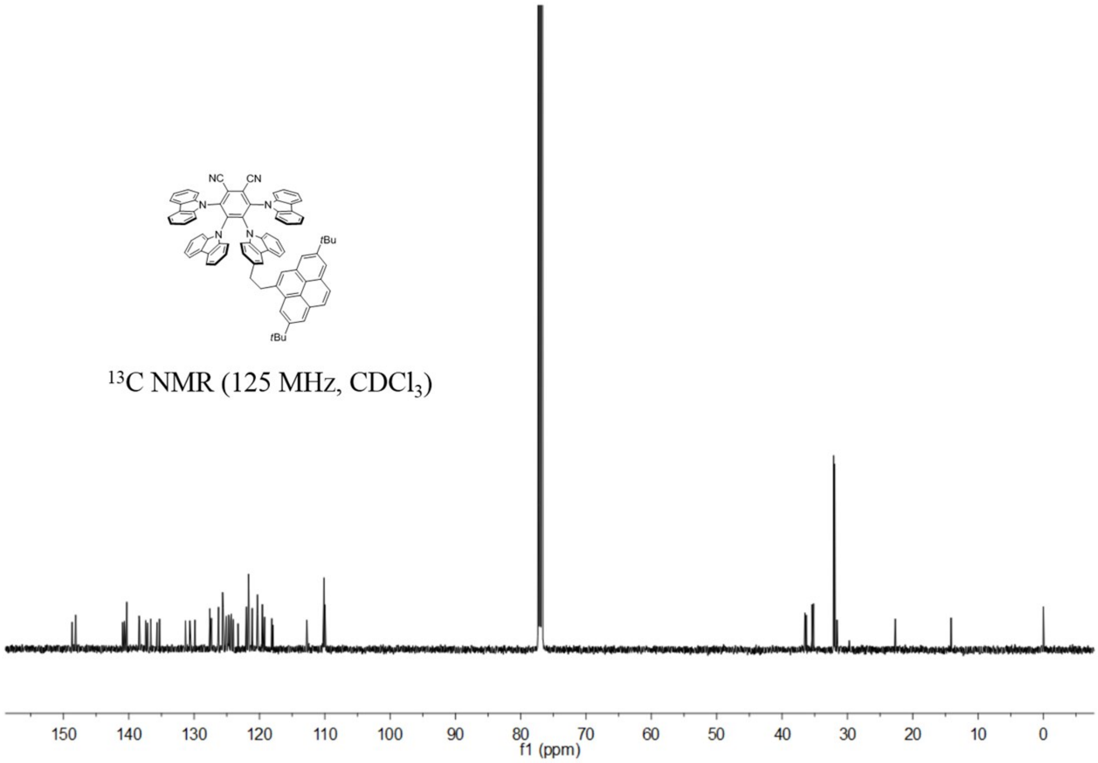

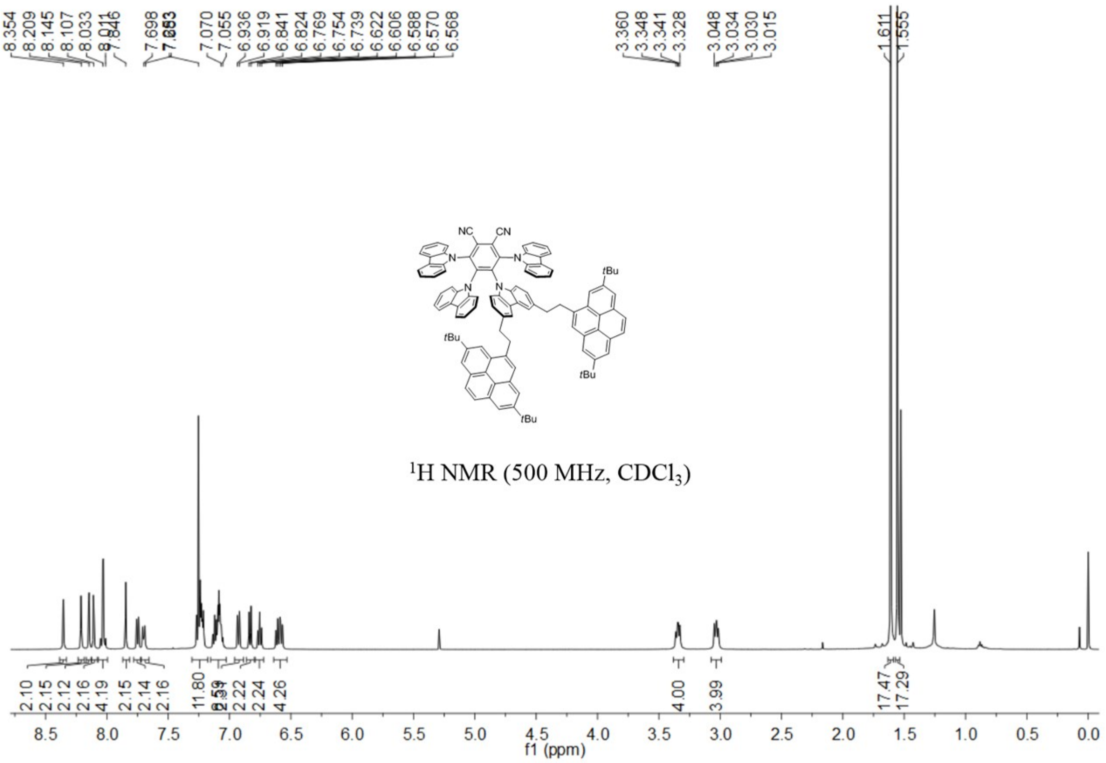
8.354
8.209
8.145
8.107
8.033
7.698
7.070
7.055
6.936
6.919
6.841
6.824
6.769
6.754
6.739
6.622
6.606
6.588
6.570
6.568
3.360
3.348
3.341
3.328
3.048
3.034
3.030
3.015
1.611
1.555
NC
CN
tBu
¹H NMR (500 MHz, CDCl₃)
2.10
2.15
2.12
2.16
4.19
2.15
2.14
2.16
11.80
2.22
2.24
4.26
4.00
3.99
17.47
17.29
f1 (ppm)

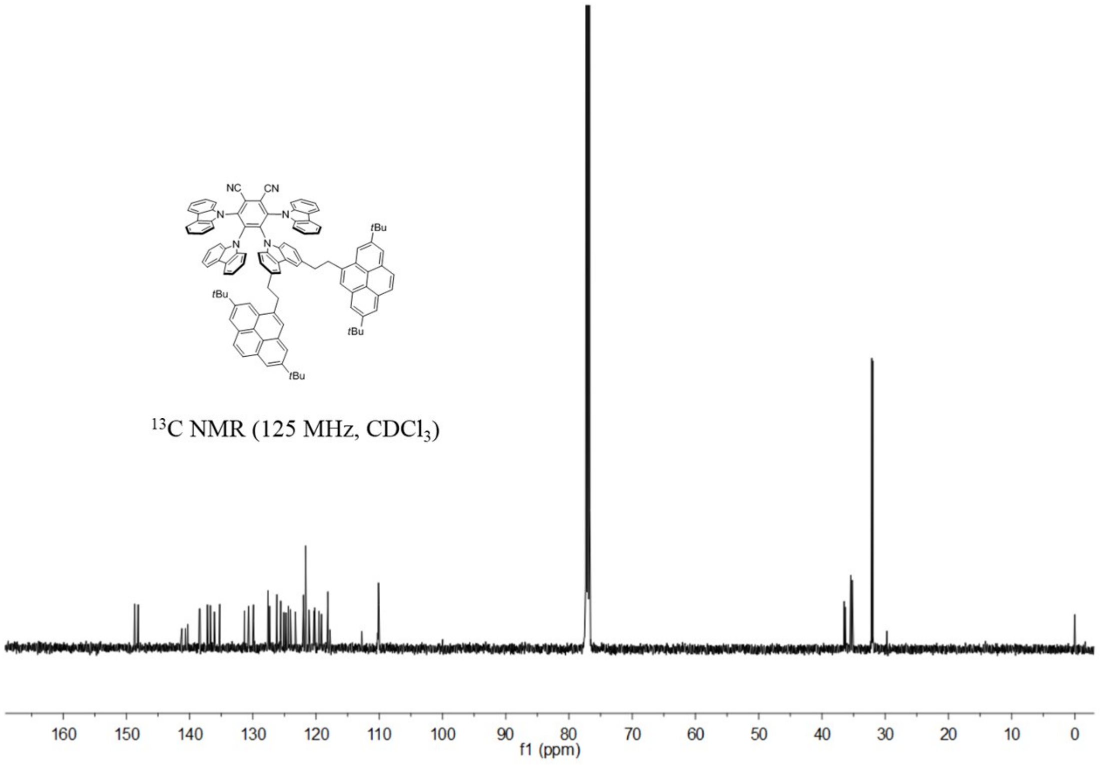
NC
CN
tBu
¹³C NMR (125 MHz, CDCl₃)
f1 (ppm)

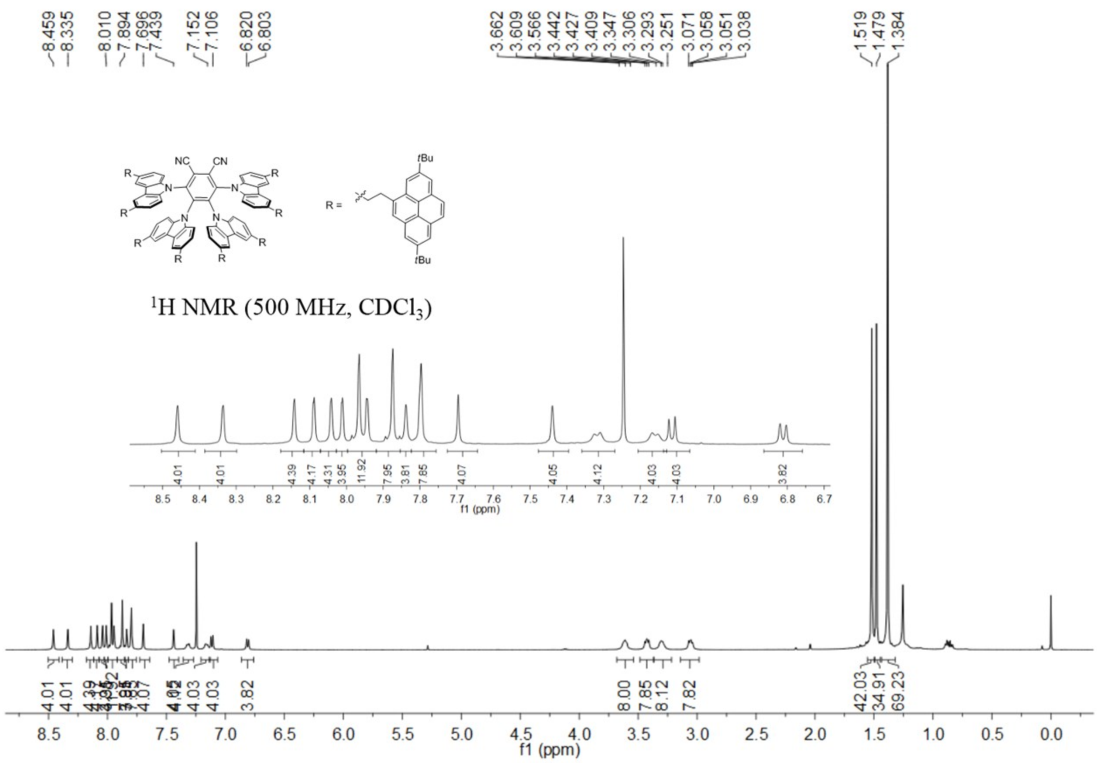

^{1}H NMR (500 MHz, $CDCl_3$)

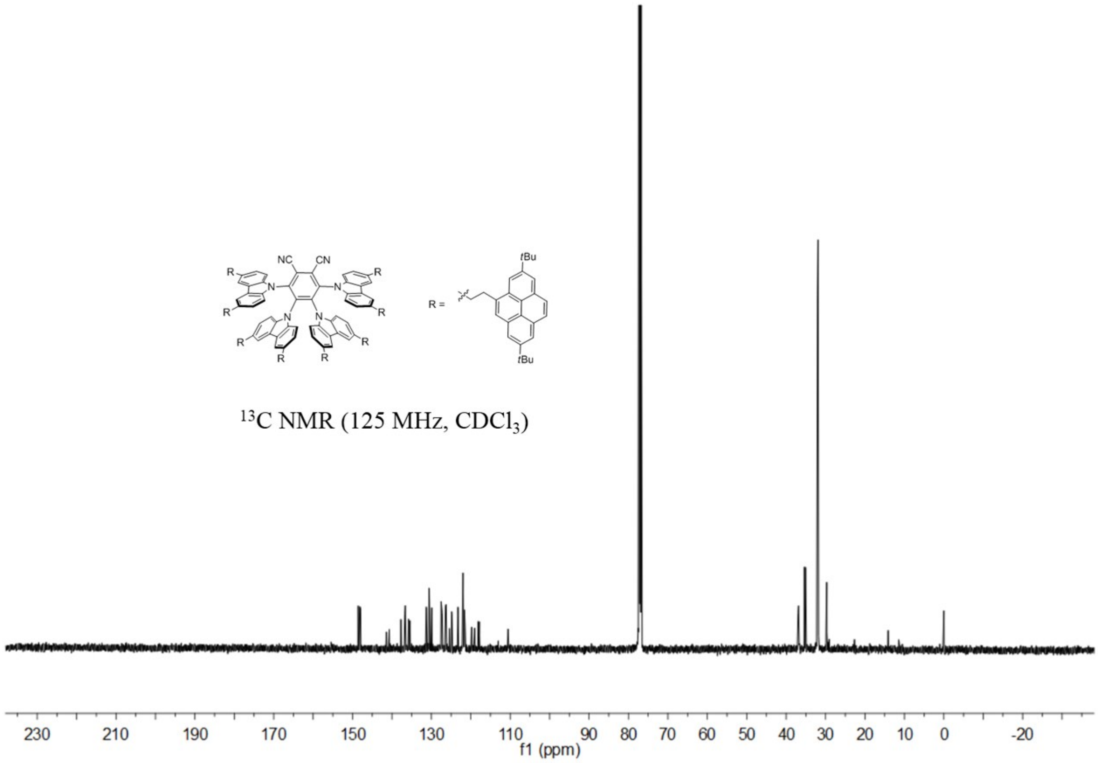

^{13}C NMR (125 MHz, $CDCl_3$)

X-ray structural analysis of 3CzFPN

Crystals for X-ray analyses of 3CzFPN were acquired by slowly evaporating a solution of the compound in DCM and petroleum ether.

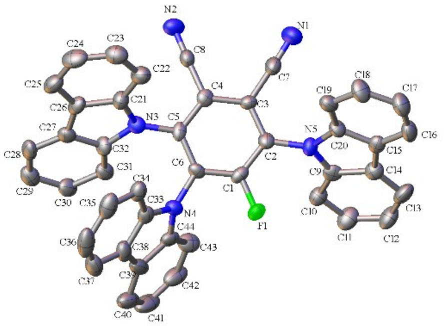

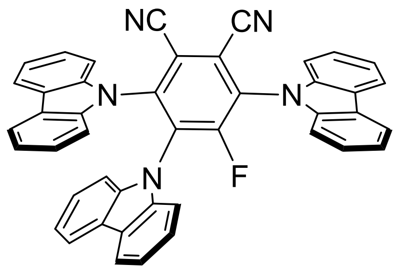

Table S1. Crystal data and structure refinement for 3CzFPN.

Identification code	**CCDC 1400097**	
Empirical formula	C45 H26 Cl2 F N5	
Formula weight	726.61	
Temperature	173.1500 K	
Wavelength	0.71073 Å	
Crystal system	Monoclinic	
Space group	P 1 21/c 1	
Unit cell dimensions	a = 11.740(5) Å	α = 90°.
	b = 21.882(9) Å	β = 92.244(8)°.
	c = 28.231(11) Å	γ = 90°.
Volume	7247(5) Å^3	
Z	8	
Density (calculated)	1.332 Mg/m^3	
Absorption coefficient	0.225 mm^{-1}	
F(000)	2992	
Crystal size	0.38 x 0.16 x 0.12 mm^3	
Theta range for data collection	1.178 to 25.200°.	
Index ranges	-14<=h<=14, -26<=k<=26, -8<=l<=33	
Reflections collected	12996	
Independent reflections	12996	
Completeness to theta = 25.242°	99.2 %	
Absorption correction	Semi-empirical from equivalents	
Max. and min. transmission	1.0000 and 0.3716	
Refinement method	Full-matrix least-squares on F^2	
Data / restraints / parameters	12996 / 74 / 986	
Goodness-of-fit on F^2	3.075	
Final R indices [I>2sigma(I)]	R1 = 0.2184, wR2 = 0.5118	
R indices (all data)	R1 = 0.2311, wR2 = 0.5154	
Extinction coefficient	0.035(4)	
Largest diff. peak and hole	1.157 and -0.983 e.Å^{-3}	
